# Maternal Health Care Among Refugees and Host Communities in Northern Uganda: Access, Quality, and Discrimination

**DOI:** 10.3389/fgwh.2021.626002

**Published:** 2021-08-20

**Authors:** Siri Aas Rustad, Helga Malmin Binningsbø, Haakon Gjerløw, Francis Mwesigye, Tony Odokonyero, Gudrun Østby

**Affiliations:** ^1^Conditions for Violence and Peace, Peace Research Institute Oslo (PRIO), Oslo, Norway; ^2^Economic Policy Research Centre (EPRC), Makerere University, Kampala, Uganda

**Keywords:** host population, integrated health system, maternal health care, quality of care, refugees, survey

## Abstract

**Introduction:** Uganda is one of the largest refugee-hosting nations in the world, with the majority of the refugees having fled South Sudan. In the early 2000's the local government and refugee health systems were merged to create a more equal and integrated system for refugees and the host population. Our aim is to investigate whether mothers from the two groups experience the same access to and quality of maternal health services, and whether refugee- and host-community mothers perceive the maternal health services differently.

**Methods:** In November–December 2019, we conducted a household survey of 1,004 Ugandan nationals and South Sudanese refugee mothers aged 15–49 in the West Nile region covering the districts of Arua, Yumbe, and Adjumani, and elicited information on access to maternal health care services, perceptions of the quality of services, and feelings of discrimination. The data was then analyzed using Ordinary Least Squares and logistic regression.

**Results:** Our analyses do not reveal large differences between refugees and the host community in terms of access to and the quality of maternal health services. Results from bivariate models indicate that refugee mothers are 6% points less likely to receive antenatal care (*p*-value < 0.05) but are 8% points more likely to give birth at a health facility (*p*-value < 0.05). Refugee mothers are generally less satisfied with how they were treated during antenatal care (0.132 lower average value on a Likert scale, *p*-value < 0.01). Refugee mothers are also 4% points more likely to feel discriminated against during ANC compared to their counterparts in the host community (*p*-value < 0.05).

**Discussion:** The way women feel treated at the health facility during maternal health care is an important aspect of quality care. While there seems to be equal access to resources between refugees and host community mothers in Northern Uganda in terms of access to and quality of care, there is still a discrepancy between the two groups in terms of how the women feel treated. Policymakers and practitioners in the health sector should pay attention to these perceived inequalities between refugees and women from the host communities to ensure equally inclusive treatment across groups.

## Introduction

In 2019, an unprecedented 70.8 million people worldwide were categorized as forcibly displaced, and the main burden of refugees and internally displaced persons falls on poor and middle-income countries. Uganda is one of the largest refugee-hosting nations in the world, with over 1.4 million refugees as of February 2020, of which the majority are refugees who fled violent conflict in South Sudan.

Uganda's remarkable approach to refugee intake makes it an especially interesting case. The country has long maintained liberal policies toward refugee intake and settlement, and more than a decade ago this practice was formalized in law. Uganda's approach to hosting refugees has been repeatedly praised as an example of best practice refugee response policy (1). With the Refugee Act of 2006, Uganda adopted legislation that granted refugees free health care within settlements, freedom of movement and to work, and the opportunity to live in refugee settlements rather than camps (2). This built on the Self-Reliance Strategy adopted in 1999, which proposed that refugees should be allocated a parcel of land to cultivate and become self-reliant rather than residing in a camp becoming dependent on support from NGOs. As a follow up, in the early 2000's, the local government and refugee health systems were merged to create a more equal, integrated system for refugees, and the host population. Before the merger, there was evidence that health facilities for refugees were better equipped and better staffed compared to health facilities for the host population in Northern Uganda, due to better financing from refugee organizations such as UNHCR compared to the Ugandan Government (3).

In this study we have chosen to focus on maternal health for two reasons: First, health care is a key determinant of human well-being, and health care services are at the heart of public goods services provided by governments. Second, maternal health is a type of health service necessary for all humans, and where knowledge about proper treatment is common to medical communities across the globe. Certain aspects of maternal health services such as antenatal care and birth delivery, are therefore comparable across otherwise different contexts.

The aim of streamlining the health care system in Uganda was to achieve more equal access to health services among refugees and the host population. However, to date, few systematic studies exploring inequalities in terms of access to health care between refugees and host populations have been carried out. Most studies of social services, such as health-care services, in developing countries focus solely on access to services, e.g., access to institutional births (4, 5). However, giving birth at a medical facility does not automatically imply high-quality health services. There can still be issues relating to, for example, expertise, waiting times, supply of medicine and other materials, and equal and fair treatment. It is not only the quality that is important but also how the woman perceives how she is treated (6).

Thus, in the present study we take a more nuanced approach to examining potential inequalities between the two groups – not only in objective terms, but also with respect to how they are perceived by the women. Our specific aims are to examine whether there are systematic inequalities between refugees and the host population in the West Nile region in terms of (1) access to maternal health services, (2) the quality of maternal health services received – in both objective and subjective terms, and (3) perceived discrimination.

## Materials and Methods

### Study Setting

Uganda has an integrated health system in which all health facilities are available for all groups of people: refugees and host population alike. However, this has not always been the case: Up until the early 2000's, the UNHCR health system in the West Nile region was operated in parallel with the publicly run service system with separate management plans and budgets (3). As a consequence of this parallel system, qualified staff such as doctors, nurses, midwives etc., moved toward the refugee health facilities, due to better pay, in addition the clinics where better equipped and staffed (7). Social services provided to refugees in Uganda have traditionally been considered better than the services offered by the districts, both by the refugees and the host population (3).

To address this apparent inequality between the refugees and the host communities, the UNHCR and the Ugandan Government developed in 1999 a Self-reliance Strategy for refugees. The aim was to empower refugees as well as nationals in the settlement areas to be able to support themselves, as well as establishing mechanisms to ensure integration. This strategy has been praised as one of the most progressive refugee policies in the world (8, 9). Consequently, the health services systems for refugees and Ugandan nationals were also integrated. Before 2000 the host and refugee populations were directed toward separate health centers, but the new integrated system allowed both groups to use any health center, and no facility was particularly geared toward refugees or nationals. This also meant that budgeting and planning became integrated and eliminated the unequal resource allocation (7). The process of merging the system started in 2000 and was finished in 2005. It is this integrated health service system that constitutes the context for our study of use and perception of maternal health services in the West Nile region in Uganda.

The West Nile region, where our survey was fielded, is home to some 750,000 refugees (or about 65% of the entire refugee population in Uganda), and in some districts refugees account for a considerable proportion of the total population, such as e.g., in Adjumani where refugees constitute ~47% of the population. The corresponding figures for the two other districts where our study took place, Yumbe and Arua, are 28 and 16%. In these districts – which are also among the poorest and least developed districts in Uganda – there are significant challenges in the provision of high-quality public services, including health services (7). The refugees from South Sudan fled an even grimmer situation, where over 20 years of armed conflict and neglect has led to poor infrastructure, little development, and a dysfunctional health system (10).

### Study Design

The data in this study was collected through a survey conducted in the three regions described above. All data were collected using tablets, and the survey was coded using Open Datakit (ODK) software. The survey interviews were conducted by South Sudanese refugees and Ugandan nationals living in the settlements, thus, they had good knowledge of the area and the various languages spoken. The enumerators underwent a 5-day training for the survey, including practice with the tablets, the ODK software, and piloting the survey. The survey was written in English, but orally translated into the local languages^1^ by the enumerators. For the questions about antenatal care and birth, women were asked to relate the questions to their most recent birth. In the sample, the years of the births range from 1991 to 2019, but about 70% of the births occurred in the three last years.

### The Study Population

**Figure 1:**
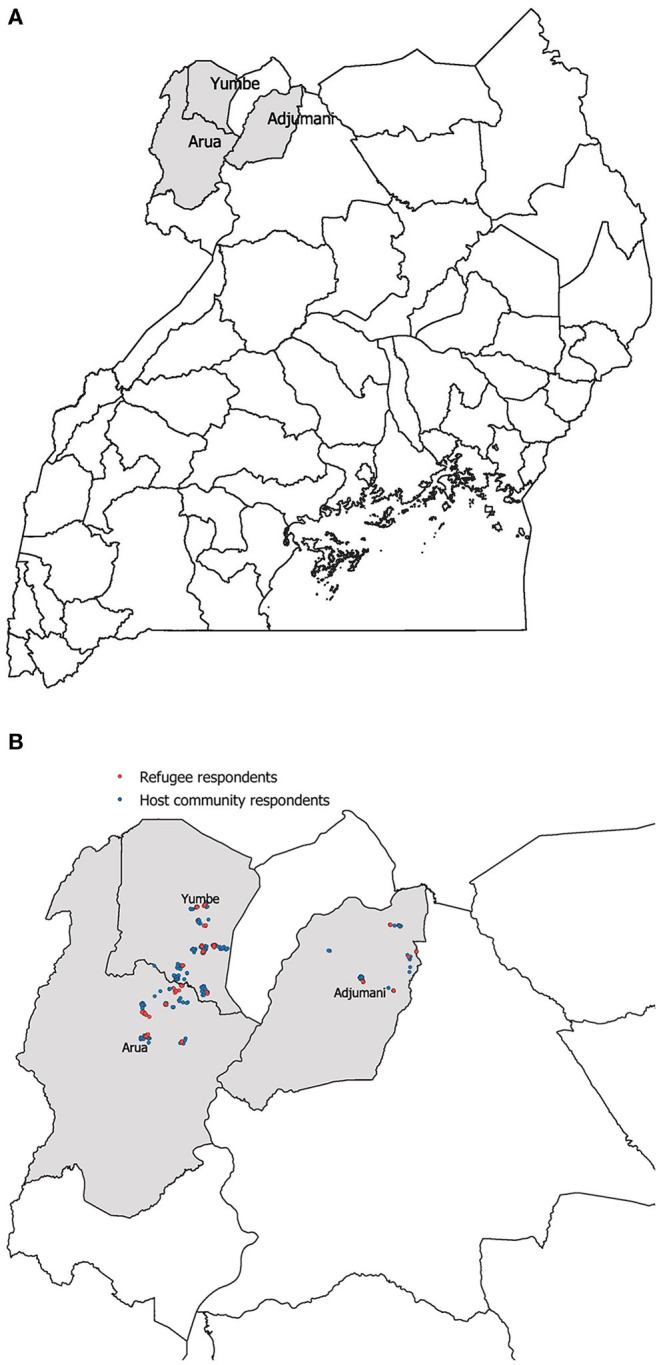
Map over districts **(A)** and geo-location of respondents **(B)**.

Our survey was conducted in November and December 2019 in three districts in Northern Uganda: Arua, Yumbe, and Adjumani (See Figure 1A). Within these districts' refugee settlements, a representative sample of 40 *enumeration areas* were randomly selected using Uganda Bureau of Statistics' sampling frame. This was done by first randomly drawing 20 refugee settlements within the three districts, and then pairing these settlements with neighboring host communities. To randomly select *respondents*, we listed all the households within all 40 enumeration areas to identify all women between 15 and 49 years living in these areas. Based on this sorting, we randomly selected 1,004 women that had given birth to at least one child^2^. We also over-sampled women with refugee status so that approximately half of the respondents were South Sudanese refugees (497), and half were respondents from the Ugandan host communities (507). The number of respondents varies from one district to the other due to size of the district: In Arua 353 respondents, in Yumbe 401 respondents, and in Adjumani 250 respondents (see Figure 1B). In cases where we were not able to reach the respondent, we replaced her with a new respondent from a randomly selected replacement list from the same district with the same host or refugee status.

### The Survey and Selected Variables

The survey used in this article was designed to investigate differences among refugees and host populations in Northwestern Uganda, specifically regarding maternal health services, but also in terms of social trust and discrimination. The questionnaire included 113 questions separated into seven modules: demographic and socio-economic status; trust and discrimination vignettes; residency and refugee status; use and access to maternal health services; perceptions of health services; feelings and experiences with discrimination; and assessment of their current situation. Most questions were developed specifically for this survey, but some were collected from other survey projects such as the Demographic and Health Surveys (DHS) Program^3^ and the EquitAble research project^4^. In this section we describe the survey questions used in this article.

Our main independent variable is a dummy for the respondent's self-reported refugee status. For the present analysis we consider a sub-sample consisting of only women who have given birth in Uganda. This sample of 841 consists of 354 refugee women and 487 host community women, equally distributed across the three districts. All the refugees in the sample are from South Sudan. The sample share of the refugee women settled in Uganda in 2016 and 2017, but also as far back as 1994.

To test the various aspects of access to and experience with maternal health care we use a range of different dependent variables measuring access to and quality of services and individual perceptions of being treated with respect, among others. We focus on antenatal care (ANC) and birth assistance.

To address our first aim of assessing whether there are differences between the refugee and host populations in terms of access to health care, we look at two indicators. The first is whether the women received ANC four times or more during their pregnancy. Four ANC visits was until recently WHO's recommended number, although this has been increased to eight in WHO's “2016 ANC model,” this includes Uganda. Nonetheless, we have chosen to use four ANC visit in this paper as only 3.3% of all respondents received 8 or more ANC checkups. We asked our sample how many times they went to ANC checkups during their pregnancy and recoded this variable to a dummy variable, taking the value 1 if the woman received ANC four or more times, and 0 if not (*antenatal care*).

As a second indicator of access to health care, we created a variable indicating whether the woman had given birth at a health facility or not. We code the variable 1 if they have given birth at a health facility, and 0 if not (*institutional birth*).

Quality of services is not only about the amount of care but also what happens during care. Our second aim is thus to examine whether there are differences in the quality of care that the two groups receive. We look at two types of quality: *Objective quality of facility* and *perceived quality of treatment*. The former is about the facility, while the latter relates to the women's experience of quality, particularly related to how she was treated.

To measure objective quality, we first asked the women whether they at least once during antenatal care had their blood pressure measured, given a urine sample, or had a blood sample taken. Based on these three variables we created an index from 0–3 indicating how many of these tests the women had taken (*antenatal tests*). We then asked the women to rate their experience of *cleanliness of the health facility* during their most recent birth on a 5-point scale from *very bad* (1) to *very good* (11) (cleanliness birth).

To capture the perception of quality, we looked at women's self-reported experiences with (1) the amount of time they waited before being attended to; (2) their experience of being treated respectfully; (3) how clearly health care providers explained things to them; and (4) their experience of being involved in making decisions for their antenatal care/birth. The women rated each experience a 5-point scale from *very bad* (1) to *very good* (11), and we created two indexes: one for antenatal care services and one for services related to giving birth *(Perceived treatment ANC/birth)*.

Finally, to address our third aim and capture perceptions of discrimination and relative deprivation, we analyze responses to the following questions from the survey: *Considering your experience with antenatal care/giving birth for your most recent pregnancy, how do you feel you were treated compared to other pregnant women?* Respondents answered on a 5-point scale from *much worse* (1) to *much better* (11). Since we are mainly interested in those who feel they were treated poorly or comparatively worse, we recoded this answer to a dummy variable where those who answered they were treated much worse or worse were coded 1 and all others coded 0 *(discriminated antenatal/birth)*. This question allows us to explore more deeply the comparative aspects between refugees and the host community.

Because we were cognizant that other factors can also affect access, quality, and discrimination with maternal health [see (6, 11)], we added several control variables that relate to the women's background and specifically about the birth. We include the women's *age* as a continuous variable. We include an ordinal variable for *level of education* 1–6: (1) no education, (2) some primary education, (3) finished primary education, (4) some secondary education, (5) finished secondary education and (6) higher education. We also include *type of employment* as a categorical variable based on women's self-reported status: (1) employed, (2) unemployed but working at home as a homemaker, farming or providing family labor, and (3) unemployed, with “employed” as the reference category. To measure wealth level, we include an asset wealth index based on questions about whether the respondents own a car, motorcycle, bicycle, radio, and/or mobile phone. We generated a linear *asset wealth index*, weighted according to their individual loading resulting from a principal component analysis. We also include the district (Arua, Yumbe, or Adjumani) of each respondent, with Arua as the reference category. Finally, we control for the most recent year of giving birth, and the number of births that the women had given in total. Descriptive statistics for the variables used in the analysis, including the control variables, are presented in Table 1.

**Table 1:** Descriptive statistics.

	**Refugee mothers**					**Host mothers**				
	***N***	**mean**	**sd**	**min**	**max**	***N***	**mean**	**sd**	**min**	**max**
**Dependent variables**										
Antenatal care	339	0.773	0.420	0	1	461	0.835	0.371	0	1
Institutional birth	341	0.944	0.230	0	1	461	0.896	0.306	0	1
Antenatal tests	325	2.606	0.652	0	3	445	2.602	0.682	0	3
Cleanliness birth	325	4.083	0.787	1	5	414	4.085	0.838	1	5
Perceived treatment ANC	318	3.575	0.617	1	5	444	3.708	0.679	1	5
Perceived treatment birth	309	3.686	0.610	1	5	406	3.800	0.681	1.250	5
Discriminated antenatal	331	0.0846	0.279	0	1	454	0.0463	0.210	0	1
Discriminated birth	327	0.0856	0.280	0	1	416	0.0745	0.263	0	1
**Control variable**										
Age	341	26.43	6.030	15	49	461	29.43	8.427	17	49
Employed	336	2.253	0.582	1	3	459	1.802	0.501	1	3
Education level	338	1.991	0.866	1	6	455	2.103	0.900	1	6
Wealth index	330	0.447	1.054	−5.403	1.361	455	−0.466	1.502	−5.403	1.361
Number of births	335	3.854	2.186	1	12	457	3.961	2.370	1	12
Birth year	339	2,018	1.509	2,003	2,019	458	2,016	4.016	1,991	2,019
District	341	1.906	0.757	1	3	461	1.920	0.772	1	3

### Analysis

The data has been analyzed by using *t*-tests to test the difference in mean between the refugee and the host mothers. Further, to test the effect of refugee status on the various maternal health indicators, we used statistical regression models. Since the various dependent variables that we test are both dummy and scale variables, we use both logit (Tables 2, 5) and OLS regression (Tables 3, 4). All dependent variables are tested in bivariate models, models with control variables, and also a model where we include an interaction between refugee status and age.

**Table 2:** Logit regression testing access to antenatal care and institutional birth and refugee status.

**Variables**	**Model 1**	**Model 2**	**Model 3**	**Model 4**	**Model 5**	**Model 6**
	**Antenatal care**	**Antenatal care**	**Antenatal care**	**Institutional birth**	**Institutional birth**	**Institutional birth**
Refugee	−0.398^**^	−0.358	0.181	0.678^**^	0.287	−3.422^**^
	(0.180)	(0.223)	(0.769)	(0.281)	(0.398)	(1.359)
Age		−0.003	0.005		−0.045	−0.076^**^
		(0.021)	(0.024)		(0.028)	(0.032)
Refugee^*^age			−0.020			0.131^***^
			(0.028)			(0.047)
Type of employment: *Ref category: Employed*						
*Working at home*		0.226	0.219		−0.602	−0.647
		(0.264)	(0.265)		(0.442)	(0.456)
*Do nothing*		−0.279	−0.270		−0.769	−0.995
		(0.334)	(0.334)		(0.632)	(0.644)
Education level		0.035	0.036		0.407^*^	0.418^*^
		(0.114)	(0.114)		(0.230)	(0.238)
Wealth index		0.016	0.015		0.099	0.094
		(0.071)	(0.071)		(0.110)	(0.112)
Number of births		0.083	0.084		−0.117	−0.127
		(0.065)	(0.065)		(0.090)	(0.093)
Birth year		0.030	0.036		0.114^***^	0.096^**^
		(0.034)	(0.035)		(0.040)	(0.041)
District: *Ref category: Arua*						
*Yumbe*		−0.469^**^	−0.471^**^		1.855^***^	1.933^***^
		(0.224)	(0.224)		(0.396)	(0.403)
*Adjumani*		−0.218	−0.222		0.991^***^	1.062^***^
		(0.265)	(0.265)		(0.373)	(0.384)
Constant	1.623^***^	−59.438	−70.756	2.152^***^	−226.972^***^	−190.115^**^
	(0.126)	(68.897)	(70.435)	(0.152)	(80.669)	(83.067)
Observations	800	754	754	802	755	755

*Standard errors in parentheses*.

*** *p < 0.01*,

** *p < 0.05*,

* *p < 0.1*.

**Table 3:** OLS regression testing the quality of services and refugee status.

**Variables**	**Model 7**	**Model 8**	**Model 9**	**Model 10**	**Model 11**	**Model 12**
	**Antenatal test**	**Antenatal test**	**Antenatal test**	**Cleanliness birth**	**Cleanliness birth**	**Cleanliness birth**
Refugee	0.004	−0.071	−0.222	−0.001	0.034	−0.241
	(0.049)	(0.058)	(0.215)	(0.060)	(0.072)	(0.266)
Age		0.004	0.002		0.006	0.003
		(0.006)	(0.006)		(0.007)	(0.008)
Refugee^*^age			0.006			0.010
			(0.008)			(0.010)
Type of employment: *Ref category: Employed*						
*Working at home*		−0.007	−0.005		0.108	0.110
		(0.068)	(0.068)		(0.086)	(0.086)
*Do nothing*		0.076	0.074		0.034	0.028
		(0.093)	(0.093)		(0.116)	(0.116)
Education level		0.029	0.029		0.024	0.024
		(0.030)	(0.030)		(0.037)	(0.037)
Wealth index		0.011	0.011		0.028	0.029
		(0.019)	(0.019)		(0.023)	(0.023)
Number of births		−0.013	−0.013		−0.027	−0.027
		(0.017)	(0.017)		(0.021)	(0.021)
Birth year		0.027^***^	0.026^***^		−0.022	−0.024^*^
		(0.010)	(0.010)		(0.014)	(0.014)
District: *Ref category: Arua*						
*Yumbe*		0.186^***^	0.186^***^		0.467^***^	0.468^***^
		(0.058)	(0.058)		(0.073)	(0.073)
*Adjumani*		−0.010	−0.009		0.287^***^	0.286^***^
		(0.067)	(0.067)		(0.085)	(0.085)
Constant	2.602^***^	−52.456^***^	−49.596^**^	4.085^***^	47.281^*^	51.562^*^
	(0.032)	(19.367)	(19.765)	(0.040)	(27.468)	(27.752)
Observations	770	724	724	739	693	693
R–squared	0.000	0.040	0.041	0.000	0.063	0.065

*** *p < 0.01*,

** *p < 0.05*,

* *p < 0.1*.

**Table 4:** OLS regression testing perceived quality of treatment and refugee status.

**Variables**	**Model 13**	**Model 14**	**Model 15**	**Model 16**	**Model 17**	**Model 18**
	**Perceived treatment ANC**	**Perceived treatment ANC**	**Perceived treatment ANC**	**Perceived treatment birth**	**Perceived treatment birth**	**Perceived treatment birth**
Refugee	−0.132^***^	−0.121^**^	−0.465^**^	−0.114^**^	−0.084	−0.150
	(0.048)	(0.057)	(0.206)	(0.049)	(0.058)	(0.214)
Age		−0.004	−0.008		−0.003	−0.004
		(0.005)	(0.006)		(0.006)	(0.006)
Refugee^*^age			0.013^*^			0.002
			(0.007)			(0.008)
Type of employment: *Ref category: Employed*						
*Working at home*		0.007	0.011		0.041	0.042
		(0.065)	(0.065)		(0.068)	(0.068)
*Do nothing*		−0.060	−0.062		0.032	0.031
		(0.089)	(0.089)		(0.092)	(0.093)
Education level		0.049^*^	0.049^*^		0.055^*^	0.055^*^
		(0.029)	(0.029)		(0.030)	(0.030)
Wealth index		−0.002	−0.002		−0.005	−0.004
		(0.018)	(0.018)		(0.019)	(0.019)
Number of births		0.002	0.002		−0.012	−0.013
		(0.016)	(0.016)		(0.017)	(0.017)
Birth year		−0.009	−0.012		−0.029^***^	−0.030^***^
		(0.009)	(0.009)		(0.011)	(0.011)
District: *Ref category: Arua*						
*Yumbe*		0.449^***^	0.450^***^		0.390^***^	0.390^***^
		(0.056)	(0.056)		(0.059)	(0.059)
*Adjumani*		0.221^***^	0.222^***^		0.216^***^	0.216^***^
		(0.064)	(0.064)		(0.068)	(0.068)
Constant	3.708^***^	20.632	26.893	3.800^***^	62.796^***^	63.845^***^
	(0.031)	(18.152)	(18.481)	(0.032)	(21.892)	(22.155)
Observations	762	721	721	715	673	673
R–squared	0.010	0.101	0.105	0.008	0.082	0.082

*** *p < 0.01*,

** *p < 0.05*,

* *p < 0.1*.

**Table 5:** Logistic regression testing discrimination and refugee status.

**Variables**	**Model 19**	**Model 20**	**Model 21**	**Model 22**	**Model 23**	**Model 24**
	**Discriminated ANC**	**Discriminated ANC**	**Discriminated ANC**	**Discriminatedbirth**	**Discriminatedbirth**	**Discriminatedbirth**
Refugee	0.645^**^	0.877^**^	3.403^**^	0.151	0.192	−0.045
	(0.298)	(0.376)	(1.346)	(0.272)	(0.332)	(1.321)
Age		−0.003	0.031		−0.010	−0.013
		(0.036)	(0.040)		(0.034)	(0.037)
Refugee^*^age			−0.095^*^			0.009
			(0.049)			(0.046)
Type of employment: *Ref category: Employed*						
*Working at home*		0.221	0.190		0.289	0.290
		(0.479)	(0.483)		(0.427)	(0.427)
*Do nothing*		0.006	0.010		−0.592	−0.597
		(0.613)	(0.625)		(0.636)	(0.636)
Education level		−0.080	−0.082		0.127	0.127
		(0.200)	(0.204)		(0.174)	(0.174)
Wealth index		−0.236^**^	−0.242^**^		0.053	0.053
		(0.118)	(0.120)		(0.117)	(0.117)
Number of births		−0.023	−0.010		0.208^**^	0.207^**^
		(0.109)	(0.112)		(0.095)	(0.094)
Birth year		−0.038	−0.009		0.046	0.043
		(0.053)	(0.057)		(0.070)	(0.071)
District: *Ref category: Arua*						
*Yumbe*		−0.928^**^	−0.939^**^		−0.761^**^	−0.759^**^
		(0.373)	(0.374)		(0.338)	(0.339)
*Adjumani*		−0.481	−0.493		−0.701^*^	−0.702^*^
		(0.407)	(0.410)		(0.391)	(0.391)
Constant	−3.026^***^	74.389	14.958	−2.519^***^	−94.911	−90.294
	(0.223)	(107.537)	(115.621)	(0.187)	(141.972)	(143.627)
Observations	785	740	740	743	697	697

*Standard errors in parentheses*.

*** *p < 0.01*,

** *p < 0.05*,

* *p < 0.1*.

### Ethical Review

In Uganda, refugee settlements are managed by the office of the Prime Minister (OPM) who are represented by the settlement commandant at the settlement level. Accordingly, the study team sought for approval from the office of the prime minister before conducting the study. Specifically, introduction letters assented to by the OPM were presented to the settlement commandants and permission had to be granted before proceeding with the field work. These letters described research activities, the kind of information to be collected, and the period of stay in the refugee settlements and host communities. In addition to the OPM approval, the respondents were presented with consent forms which they either signed or finger printed for those who could not read and write. For women aged 15–17 years, the consent was ascertained from their legal guardians. The sampling frame was provided by the Uganda Bureau of Statistics (UBoS), an official government body mandated with the design, and generation of national official statistics. UBoS has divided the country into enumeration areas (EAs) and the study used the frame to randomly sample 40 EAs. The survey was implemented by the Economic Policy Research Centre (EPRC), based at Makerere University.

Before the interview started, the enumerators asked for the respondent's consent, explained the purpose and initiators of the study, their legal right not to participate, and ensured the respondent full anonymity and confidentiality. The respondents were presented with the full consent form, but the details were also verbally communicated. The data collection is also registered with the Norwegian Center for Research Data. This includes, among other things, an ethical review of the content of the survey, its consent forms, and procedures for secure data storage.

While we did not expect participation in the study would entail any harm or risk of (re-)traumatization for the respondents, the training included a section where enumerators were prepared to face emotional reactions to the survey questions. In addition, all the questions were closely discussed with the enumerators to make sure the wording was not insulting or problematic to talk about.

## Results

### Access to Services

To address our first aim, we look at the distribution of our dependent variables. Out of the 846 women in our sample 165 or 19.5% of the women had been to checkups less than four times. When we break it down by group, there is a clear difference between refugees and hosts, 77 out of 339 (22.7%) refugee mothers, and 76 out of 461 (16.5%) host mothers. A simple *t*-test confirms significant difference between the two groups. Further, about 92% of the sample had given birth at a health facility. When we break it down, we see that there is a large discrepancy between the host population and refugees: 10.5% and 5.5% of the host population and refugees, respectively, did not give birth at a medical facility. A *t*-test indicates that the difference is just above a 0.1 level of significance.

With respect to whether a woman has received at least the four recommended number of ANC checkups during pregnancy, we find the refugee variable to be negative [Coef −0.398, SD 0.180, *p* < 0.05 (bivariate) [Coef −0.358, SD 0.223, *p* > 0.1 (with controls)] in both the bivariate and multivariate models (Table 2, models 1 and 2). This suggests that refugee mothers receive fewer ANC checkups than host mothers. However, the result is only significant in the bivariate model. There does not seem to be any effect of age on this (Table 2 Model 3).

**Figure 2:**
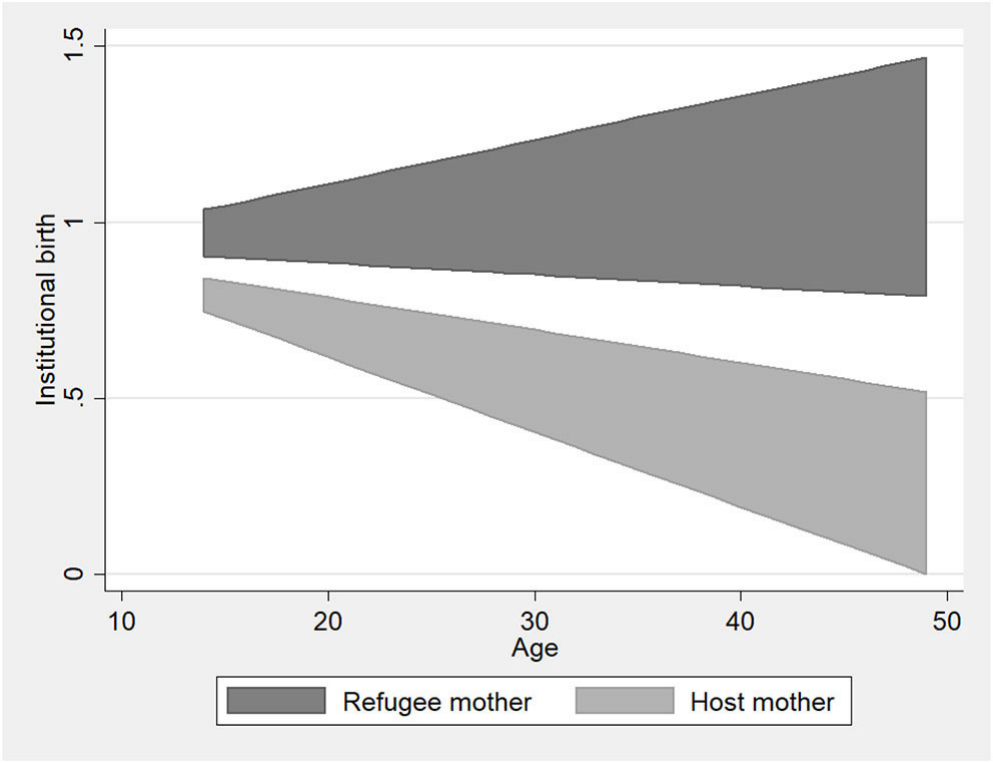
Relationship between institutional birth and the Interaction between refugee status and age (based on Model 6).

In contrast to the analysis on ANC checkups, the birth at a medical facility analysis (institutional birth) shows the refugee variable to be positive and significant [Coef 0.678, SD 0.281, *p* < 0.05 (bivariate)] (Table 2, models 4 and 5). This suggests that refugee mothers more often give birth at a medical facility compared to host community mothers. However, the significance disappears when we add control variables [Coef 0.287, SD 0.398, *p* > 0.1 (with controls)]. When asked why they did not give birth at a medical facility, 50 out of 77 respondents cite challenges with accessibility (e.g., lack of company, lack of transportation, distance, no service, or accessibility), and there was no difference in how refugee and host community mothers responded. The inclusion of the interaction term further indicates that for refugee mothers the likelihood of giving birth at a medical facility do not change substantially with age, while for host mothers it decreases with age (Table 2 Model 6, also see Figure 2). One explanation for this can be that the analysis only includes births that have happened in Uganda, and thus, the included refugee might have given birth more recently than the host mothers and thus benefited by a better health system overall. The median birth year (Table 1) supports this as the median for refugee mothers is 2018 and for host mothers it is 2016.

### Quality of Services

We now turn to the *quality* of maternal health care services. As mentioned above, mere access to health services will not necessarily ensure good quality health care. However, quality of care is a decisive factor in reducing the number of maternal deaths (6).

### Availability of Medical Tests and Cleanliness of Facilities

We test objective quality of the medical facility by looking at the total number of standard tests a pregnant woman took during ANC (e.g., blood pressure, urine sample, and blood sample) and the cleanliness of the birth facility. The descriptive data (Table 1) reveal that the vast majority – ~70% – had taken all three, that is 536 out of 770. The number for refugees were 224 out of 325 (69%) and 312 out of 445 (70%). Nonetheless, nine women had not taken any, and 234 women (~30%) did not receive all these simple, but important tests. Most respondents rated the cleanliness as good or very good, 621 out of 739 (more than 80%), and only 30 (4%) found it to be bad or very bad. However, the differences between hosts and refugees seem marginal. This impression is confirmed in bivariate and multivariate analyses (Table 3).

For the refugee variable, the results are inconsistent from the bivariate models to the models with control variables for both dependent variables, and it is insignificant in all six models in Table 3 [Coef 0.004, SD 0.049, *p* > 0.1 (bivariate); Coef −0.071, SD 0.058, *p* > 0.1 (with controls); Coef −0.001, SD 0.060, *p* > 0.1 (bivariate); Coef 0.034, SD 0.072, *p* > 0.1 (with controls)]. This indicates that there is little systematic difference between refugee and host-community mothers when it comes to reported objective quality. Age does not have any effect on these results (Table 3, Models 9 and 12).

### Perceived Quality of Treatment

**Figure 3:**
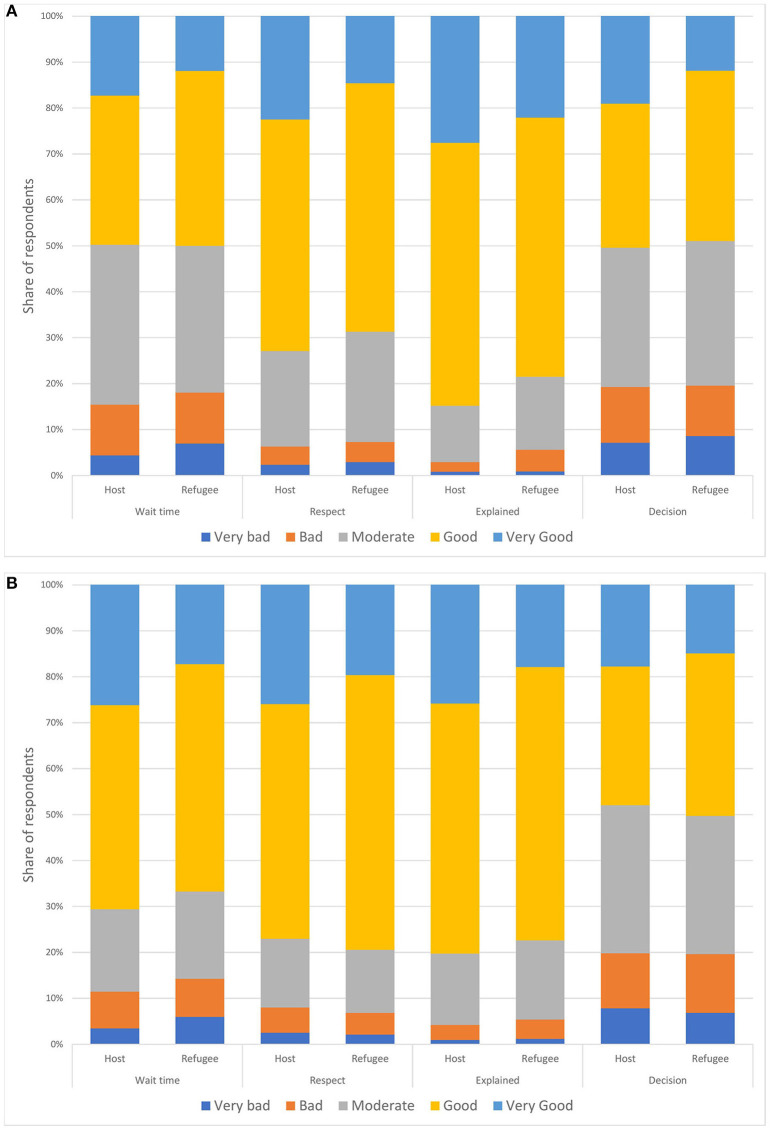
Perceived quality of treatment by refugee status. Antenatal care in **(A)** and birth care in **(B)**.

In contrast to the objective measures where there were marginal differences between the groups, we found larger differences when we examined the perceived quality of treatment. Although, the vast majority of all respondents were quite satisfied with the level of treatment overall, there is quite a bit of variation within each of the variables that the index is constructed from, particularly for the variables measuring wait time and ability to affect decisions. For these two categories, ~20% of all respondents answered bad or very bad. Comparing refugee women to host community women we see that the latter is more likely to be more satisfied. For some of the variables, the bad and very bad categories are slightly higher for refugees compared to hosts (Figure 3).

We created indexes for measuring the perceived quality of treatment for both ANC and birth assistance based on these factors. We find that 108 out 444 (24%) host mothers scored above four on the index for ANC checkups, the corresponding number for refugees where 38 out of 318 (12%), only nine women in total scored <2 on the index. For the index on birth assistance 110 of 432 (27%) host mothers and 51 out of 309 (16.5%) refugee mothers scored more than four, while 12 women in total scored <2.

The analyses of the indexes show that the refugee status variable is negative in all the models, suggesting that refugee mothers feel less respected and welcomed than host community mothers (Table 4). For ANC, the variable is significant both in the bivariate analysis and with control variables [Coef −0.132, SD 0.048, *p* < 0.01 (bivariate); Coef −0.121, SD 0.057, *p* < 0.05 (with controls)]. The interaction with age is significant [Coef 0.013, SD 0.007, *p* < 0.1], however further scrutinizing of this indicates that the effect is very small. For perceived treatment at birth only the bivariate analysis is significant [Coef −0.114, SD 0.049, *p* < 0.05 (bivariate)], but the *p*-value for the refugee variable in the analysis with control variables is close to an acceptable significant level [Coef −0.084, SD 0.058, *p* = 0.15 (with controls)]. Age has no effect on perceived treatment at birth.

### Perceived Discrimination in Treatment

Finally, we test how refugee status affects the perception of being discriminated. We find that 28 out of 331 (8.5%) of the refugees felt they were treated worse than others during antenatal care, while only 21 out of 454 (4.5%) among the host population felt the same. During delivery 28 out of 327 (8.5%) of the refugees and 31 out of 416 (7.5%) of the host women felt treated worse than others. In all the models in Table 4, the refugee variable is positive, suggesting that refugee mothers feel treated worse than others, but the result is only significant when testing ANC [Coef 0.665, SD 0.298, *p* < 0.05 (bivariate); Coef 0.877, SD 0.376, *p* < 0.05 (with controls); Coef 0.151, SD 0.272, *p* > 0.1 (bivariate); Coef 0.192, SD 0.332, *p* > 0.1 (with controls)]. When looking at the substantial effects of this finding, we see the risk of feeling discriminated against during ANC increases from 4% among the host population to 10% among the refugee women. Mothers who answered that they were treated worse or much worse were also asked why they felt this way. Among the 29 refugee mothers who answer this way, 20 responded it was due to their refugee status or language. For giving birth, 18 out of the 29 who felt treated worse mentioned the same reasons. Among the host community mothers, the reasons for feeling treated worse were more diverse. The analysis also suggests that this effect increases with age, but the effect of the interaction term is very small [Coef −0.095, SD 0.049, *p* < 0.1].

For the control variables in the analyses we find varying results, and it is difficult to draw any firm conclusions. However, across the models, education level seems to be the most stable variable, with primarily a positive effect associated with access [Coef 0.407, SD 0.230, *p* < 0.1 (Table 2, Model 6)], quality [Coef 0.024, SD 0.037, *p* > 0.1 (Table 3, Model 12)], and perceived quality of maternal health services [Coef 0.055, SD 0.033, *p* < 0.1 (Table 4, Model 18)]. This finding corresponds to a similar finding in Saifuddin (11). Employment seems to matter for the perception of the health facility, where mothers doing nothing or working from home are more approving of maternal health services than those who are employed and working out of the house. Wealth seems to matter for whether a mother felt discriminated against [Coef −0.236, SD 0.118, *p* < 0.05 (Table 5, Model 20)]. For age, birth year and number of births, the effects are very small, and mostly insignificant.

Interestingly, we find large differences among the three districts (Arua, Yumbe, and Adjumani). In most cases, mothers from Yumbe report better quality [Coef 0.467, SD 0.073, *p* < 0.01 (Table 3, Model 8)] better perceived quality [Coef 0.390, SD 0.059, *p* < 0.01 (Table 4, Model 18)], and less discrimination [Coef −0.761, SD 0.338, *p* < 0.05 (Table 5, Model 23)] compared to those from Arua. This finding is significant in most models. We see a similar trend for Adjumani, but the results are less strong [Coef 0.287, SD 0.085, *p* < 0.01 (Table 3, Model 12); Coef 0.216, SD 0.068, *p* < 0.01 (Table 4, Model 12); Coef −0.701, SD 0.338, *p* < 0.1 (Table 5, Model 16)]. While Arua, in general, is more developed than the two other districts, these results could be explained by the fact that the refugee settlements in Arua are further away from Arua town, while the refugee settlements in Yumbe and Adjumani are closer to main towns in Yumbe and Adjumani. In addition, the roads to Imvepi and Rhino Camp in Arua are in poor condition, while in Yumbe and Adjumani the refugee settlements are situated close to the main roads, both in good condition.

## Discussion

Refugees are a particularly vulnerable group when it comes to health. Violent conflicts have often forced them to leave their homes and flee to neighboring countries, exposing them to numerous challenges that affect their health such as lack of food, lack of clean drinking water, danger of being captured, and sexual violence. In addition, the psychological trauma of persecution and leaving family behind is extreme (12). With a sudden influx of refugees into a developing country, the burden on an often-already fragile health system is enormous. Refugee camps, mostly run by NGOs and coordinated by UNHCR to deal with immediate health issues, are often overcrowded and set up for emergencies, and not for long-term health services (13). Previous research has shown that refugees are likely to have increased morbidity, poor health habits and a decreased life expectancy (14). Most existing research looks either at how forced migration affects refugees' access to health services, or the effect of living close to a refugee camp on the host population. Very few studies have looked at the differences in health services comparing host population and refugees living in the same area, even though more research on planning, delivery, and utilization of health services for host and refugee populations is clearly needed, particularly in situations of long-term displacement [(15) pp 241].

Weiss et al. (16) provide one of the few studies grappling with such a comparison. They compare utilization of health services among refugees and host populations in 102 refugee sites in 25 countries in Asia, Africa, and the Middle East. They found that between 2008 and 2009, the host populations were much less likely to visit health facilities in refugee sites than refugees themselves, even though the health facilities were open to nationals as well. They suggest this difference is due primarily to government health facilities being closer to these host communities than the refugee settlements. While Weiss et al. 's study on refugee sites found a lower rate of utilization of the refugee health facilities among the host population in general, their specific results for Uganda (16) indicated an opposite trend: that the host population used the services more than the refugees. They argue this is due to the integrated health system in Uganda but call for more research to assess the quality of the services.

In response to this call, this study compares the experiences of mothers from the refugee and host communities in the West Nile region in Uganda regarding access to and quality of maternal health care received – in both objective terms and in terms of how the mothers perceived the quality and potential discrimination during care. The Uganda case presents a unique opportunity to compare the experiences of refugees to the host community because since early 2000, health services in the region have been integrated so that both populations are treated by the same health care system. Moreover, through our approach of directly surveying the refugees and host population, we have been better able to capture how mothers perceive the quality of the health care they have received compared to studies such as Weiss et al. (16) that have relied on aggregate data at the refugee settlement level from UNHCR's Health Information System (including numbers on settlement population, outpatient health services, diagnoses, etc.,).

Our analyses do not reveal any particularly large differences between refugees and the host community when it comes to the access to and quality of maternal health services. While we do find some differences between the two groups, they are relatively small. However, this might not be very surprising exactly because the health system in Uganda is integrated, where the refugees and the host community use the same facilities. This corresponds to findings by (7) showing that before the integration of the health system, health facilities for refugees were better equipped and staffed compared to those used by the host population. There might well be larger differences between refugee and host populations in other areas of Uganda than the West Nile because according to the DHS data, mothers in the West Nile region enjoy better access to and quality of maternal health care compared to the rest of Uganda. However, despite the relatively better conditions in this area, there is still clear room for improvement, as 30% of all the women in our sample did not receive a blood pressure check, nor provide a urine sample and blood sample during antenatal care. These easy, but important, tests should be standard for antenatal care providers.

Findings from other settings have not been consistent on whether significant differences exist in the provision of care between refugee and host populations. One study of reproductive health indicators in UNHCR post-emergency camps between 2007 and 2013 shows there has been improvement over time, and that many of the reproductive indicators for refugees mark higher compared to the host country recipients (17), suggesting that refugee women receive relatively better care than the host population. On the other hand, a study of the Rohingya refugee crisis in Bangladesh, shows that the water, sanitation, and hygiene services reached only 30% of the Rohingya refugees, and that only 22% of pregnant Rohingya women gave birth at medical facilities (18), suggesting that in this case refugees received worse care than the host population. This is contrary to what we find in our study, where as many as 94% of the refugees gave birth at a medical facility.

Although, we found few differences between the refugees and the host community in terms of their access to health care and the quality of health care received, we found that refugee mothers tend to view themselves as being treated worse than host mothers. The perceived difference between refugee mothers could be related to expectation and previous experiences, but it could also indicate that refugee mothers are treated differently or discriminated against. Discrimination during care, beyond tangible acts such as retention of services, is challenging to study. However, it is reasonable to assume that perceived discrimination is likely to affect trust in the host community and its services, as well as the likelihood that one will seek health care when needed.

Our approach of comparing the experiences of refugees to the host communities, rather than to some objective standard such as the UNHCR standards (17), has allowed us to better capture the quality of services relative to what can be expected in the local context. For example, if the UNHCR standards are set too high, countries with lower GDP per capita or inadequate infrastructure, for example, could fail to meet the standard even if the displaced communities were extensively prioritized in national budgets. By comparing with the host communities, we were able to obtain a relative scale for comparison which showed that although there might be room for improvement on an absolute scale, in relative terms refugee populations are receiving care that is equivalent to host populations. However, such comparisons with the host community have two important caveats. First, there might be an *adverse selection* in the settlement of refugees. The West Nile region in Uganda is less developed than the southern parts of the country. Second, refugee influx might affect the quality of services provided to the host community. If so, the comparison is a moving target.

## Conclusions

Uganda has the highest number of refugees among all African countries. The bulk of the refugees come from neighboring countries, reflecting the unstable politics in the region. The West Nile region, where our survey was fielded, is home to some 750,000 refugees (or approximately 65% of the entire refugee population in Uganda). In this region, we find some of the poorest and least developed districts in Uganda, with significant challenges in the provision of high-quality public services, including health services. Refugees from South Sudan fled an even grimmer situation, where more than 20 years of armed conflict and neglect has led to poor infrastructure, little development, and a dysfunctional health system (16). Compared to most other refugee-hosting countries – where refugees live in camps separated from the host population – refugees in Uganda live in settlements.

While the health system in Uganda is integrated and should provide equal access to resources and opportunities among refugees and host population, there are tensions between groups. In September 2020, at least ten people were killed in Rhino Camp (one of the target areas of our survey) due to a quarrel over water resources between the refugees and host community^5^. This type of tension can lead to suspicion, ill treatment of certain groups and discrimination. Our findings allude to this. The results from the present study indicate that while there appears to be equal access to maternal care resources for refugees and host community mothers in Northern Uganda, there are still differences between the two groups in terms of how the women feel treated, with refugee mothers reporting a higher level of perceived discrimination. Most of the refugee women who feel discriminated against argue this is due to their refugee status or use of a different language.

The way women feel treated at the health facility during maternal health care is an important aspect of quality care (6). This includes trust, confidentiality, social support, and respectful communication. And these elements are arguably especially important for vulnerable subgroups such as refugees. Policymakers and practitioners in the health sector should be attentive to perceived inequalities between refugees and women from the host communities to ensure feelings of inclusive treatment across groups. Not only is inclusive treatment a goal for public health itself, but it may also be an effective investment in peaceful intergroup relations.

Our study may be useful for medical personnel, aid workers, public health officials, and government officials. However, our findings need to be interpreted with caution and considered within the context of several limitations. First, our study is cross-sectional. Future studies should strive to obtain temporal data to assess the impact of particular interventions and health reforms when it comes to improving access, quality and inclusiveness of maternal health care. Second, our sample was limited to a single country, thus, findings should only be interpreted within the context of South Sudanese refugees and Ugandan women. Future studies should explore the same questions in other contexts where refugees and host communities reside side by side. Third, our measures of quality of health care are quite crude. Future research should try to devise more robust, nuanced measures that distinguish between the objective quality of medical institutions, personnel, and care.

Despite the mentioned limitations, we believe our findings remain valuable for developing evidence-based policy advice and for informing and inspiring more research on this pressing concern for public health.

## Data Availability Statement

The data analyzed in this study is subject to the following licenses/restrictions: the data can be provided upon request and will be published when the article is published. Requests to access these datasets should be directed to Siri Aas Rustad, sirir@prio.org.

## Ethics Statement

The studies involving human participants were reviewed and approved by Norsk Senter for Forskningsdata and the office of the prime minister in Uganda. Written informed consent to participate in this study was provided by the participants' legal guardian/next of kin.

## Author Contributions

SR has been main responsible for the article. GØ and HB has contributed to the writing and analysis. SR, HB, HG, FM, and TO were responsible for collecting the data.

## Conflict of Interest

The authors declare that the research was conducted in the absence of any commercial or financial relationships that could be construed as a potential conflict of interest.

## Publisher's Note

All claims expressed in this article are solely those of the authors and do not necessarily represent those of their affiliated organizations, or those of the publisher, the editors and the reviewers. Any product that may be evaluated in this article, or claim that may be made by its manufacturer, is not guaranteed or endorsed by the publisher.

## Footnotes

^1^The most common languages among the local population are Madi and Lugbara. Among the refugee population the most common languages are Kakwa, Pojulu, Bari, Madi, Dinka.

^2^All identifiable information was deleted after the survey interview was conducted.

^3^ https://dhsprogram.com/

^4^Enabling Universal and Equitable Access to Healthcare for Vulnerable People in Resource Poor Settings in Africa: https://www.sintef.no/projectweb/equitable/.

^5^ https://www.voanews.com/africa/unhcr-urges-investigations-after-10-refugees-killed-uganda-water-conflict
